# Mitochondrial dysfunction and therapeutic perspectives in osteoporosis

**DOI:** 10.3389/fendo.2024.1325317

**Published:** 2024-02-02

**Authors:** Jialing Liu, Zhonghua Gao, Xiangjie Liu

**Affiliations:** ^1^ Department of Geriatrics, Liyuan Hospital, Tongji Medical College, Huazhong University of Science and Technology, Wuhan, China; ^2^ School of Medicine, Ezhou Vocational University, Ezhou, China

**Keywords:** osteoporosis, mitochondrial dysfunction, oxidative phosphorylation, reactive oxygen species, mitochondrial quality control

## Abstract

Osteoporosis (OP) is a systemic skeletal disorder characterized by reduced bone mass and structural deterioration of bone tissue, resulting in heightened vulnerability to fractures due to increased bone fragility. This condition primarily arises from an imbalance between the processes of bone resorption and formation. Mitochondrial dysfunction has been reported to potentially constitute one of the most crucial mechanisms influencing the pathogenesis of osteoporosis. In essence, mitochondria play a crucial role in maintaining the delicate equilibrium between bone formation and resorption, thereby ensuring optimal skeletal health. Nevertheless, disruption of this delicate balance can arise as a consequence of mitochondrial dysfunction. In dysfunctional mitochondria, the mitochondrial electron transport chain (ETC) becomes uncoupled, resulting in reduced ATP synthesis and increased generation of reactive oxygen species (ROS). Reinforcement of mitochondrial dysfunction is further exacerbated by the accumulation of aberrant mitochondria. In this review, we investigated and analyzed the correlation between mitochondrial dysfunction, encompassing mitochondrial DNA (mtDNA) alterations, oxidative phosphorylation (OXPHOS) impairment, mitophagy dysregulation, defects in mitochondrial biogenesis and dynamics, as well as excessive ROS accumulation, with regards to OP (**Figure 1**). Furthermore, we explore prospective strategies currently available for modulating mitochondria to ameliorate osteoporosis. Undoubtedly, certain therapeutic strategies still require further investigation to ensure their safety and efficacy as clinical treatments. However, from a mitochondrial perspective, the potential for establishing effective and safe therapeutic approaches for osteoporosis appears promising.

## 1 Introduction

Osteoporosis is a highly prevalent metabolic bone disease worldwide, affecting over 20 billion individuals globally. Among them, approximately 50% of women and 20% of men will experience an osteoporotic fracture after the age of 50 years (1, 2). Notably, due to an aging population and increasing life expectancy, it is projected that the number of individuals aged 50 years and older with osteoporosis will surpass 400 million by 2050 (3). Osteoporosis has emerged as a significant and escalating public health concern owing to its widespread occurrence and the severe implications associated with osteoporotic fractures (4). Regrettably, despite extensive research efforts, the precise pathogenesis underlying osteoporosis remains elusive while effective interventions are still lacking.

**Figure 1:**
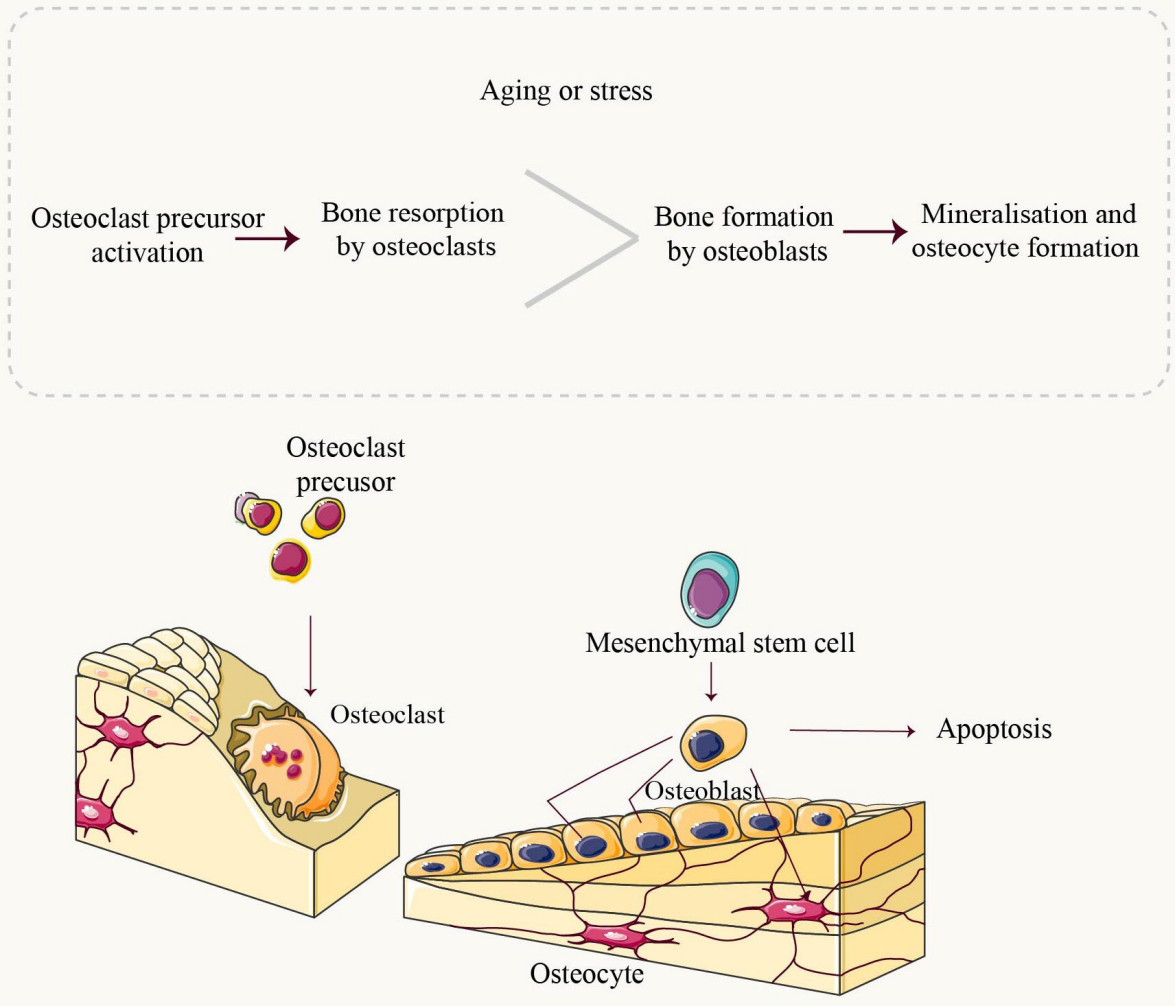
Mitochondrial dysfunction and osteoporosis. Mitochondria are double-membrane organelles found in eukaryotic cells, playing a pivotal role not only in ATP production through oxidative phosphorylation but also in apoptosis and autophagy. Under physiological conditions, Mitochondria quality control involves mitochondrial fusion and fission, biogenesis, mitophagy, as well as antioxidant defense systems to establish a mitochondrial network that can governs mitochondria homeostasis and ensuring normal cellular function. When under conditions of stress or aging, an imbalance in this control system can lead to the production of abnormally functioning mitochondria. Mitochondrial dysfunction is primarily characterized by alterations in mitochondrial DNA (mtDNA), impaired oxidative phosphorylation (OXPHOS), disruptions in mitochondrial autophagy, defects in mitochondrial biogenesis and dynamics, and excessive accumulation of reactive oxygen species (ROS). Disruption of mitochondrial function leads to impaired homeostasis, resulting in intracellular disorders or cellular dysfunction that ultimately impacts bone homeostasis and induces the onset and progression of osteoporosis.

Mitochondria, complex organelles present in all cells except erythrocytes, are typically located in the cytoplasm and often adjacent to the endoplasmic reticulum (ER) (5). They play a crucial role in various cellular processes, particularly adenosine triphosphate production (6). The process of normal adult bone growth is characterized by a delicate equilibrium between bone resorption and bone formation. Osteoclasts (OCs), located on the surface of dissolved bone, break down existing bone into its fundamental components (primarily type I collagen and inorganic salts), leading to bone resorption. On the other hand, osteoblasts (OBs), situated on the surface of new bone, utilize these broken-down components provided by osteoclasts to generate a fresh matrix for subsequent mineralization (**Figure 1**). This newly formed matrix is then mineralized by osteoblasts that become entrapped within it and eventually transform into osteocytes. Both osteoclasts and osteoblasts originate from the marrow cavity within bones (7–9). The involvement of mitochondria in bone is crucial for the regulation of nutrient metabolism and maintenance of bone homeostasis (9, 10). Numerous studies have consistently demonstrated that mitochondrial dysfunction is both a causative factor and an indicative symptom of the aging process, owing to the inevitable impairment of cellular function (7, 11). Mitochondrial dysfunction is now recognized as a pivotal factor in the mechanisms underlying aging and has been demonstrated to be a prevalent characteristic associated with age-related diseases (9).

Notably, it is now tentatively suggested that mitochondrial dysfunction in bone cells may contribute to osteoporosis by disrupting the normal activity of bone cells and impairing their function in maintaining bone homeostasis (12). Currently, only a limited number of studies have elucidated the association between osteoporosis and mitochondrial dysfunction (13–15).Therefore, this paper comprehensively reviews diverse manifestations of mitochondrial dysfunctions in osteoporosis and further investigates the potential therapeutic strategy of modulating mitochondrial function for OP treatment.

## 2 Mitochondria: structure and function

Mitochondria, the primary source of metabolic energy in aerobic eukaryotic cells, play a pivotal role in generating ATP through OXPHOS (16). Apart from their fundamental function in energy provision, mitochondria are indispensable for regulating cell cycle progression and programmed cell death (17). Furthermore, mitochondria govern various metabolic processes including calcium signaling, the citric acid cycle, amino acid metabolism, and phospholipid biosynthesis (18).

To fulfill these essential functions, mitochondria possess the capacity to modulate their quantity and dimensions in response to cellular metabolic activity (19). Consequently, mitochondria exhibit remarkable dynamism by undergoing fission and fusion events, enabling the formation of interconnected tubular networks (20). Furthermore, mitochondria are semi-autonomous organelles harboring their genetic material (21). Mitochondrial DNA has a circular structure and comprises 16,569 base pairs containing 37 genes encoding 13 mitochondrial proteins, 22 transfer RNAs, and 2 ribosomal RNAs (22). These proteins encoded by mtDNA constitute an integral component of the electron transport chain complex’s central subunit (23). Moreover, over 1000 additional proteins are synthesized from nuclear DNA and subsequently transported across the cytoplasm to fulfill mitochondrial functions (24). The maintenance of normal respiratory chain activity necessitates an intact and functional mitochondrial genome (25). Coordinated interactions between nuclear DNA and mtDNA are indispensable for the preservation of optimal mitochondrial function (26).

Mitochondria are enclosed by a double phospholipid membrane, effectively separating them from the cytoplasm. The outer mitochondrial membrane exhibits porosity and relatively high permeability, lacking a membrane potential. In contrast, the inner mitochondrial membrane functions as a selective diffusion barrier responsible for cellular energy production; its ion selectivity establishes a potential difference across this membrane (27). The ETC, consisting of five complexes [CI-CV], coenzyme Q10 (CoQ10), and cytochrome c, is localized on the inner mitochondrial membrane (28). Energy derived from the process of oxidative phosphorylation through the respiratory chain is utilized for proton pumping, resulting in the establishment of an electrochemical gradient across the inner interstitial space and mitochondrial matrix (29). These proton movements facilitate ATP synthesis via complex V (ATP synthase). Reactive oxygen species, including free radicals and hydrogen peroxide, are also generated during oxidative phosphorylation as by-products (30, 31). These ROS can cause damage to lipids, nucleic acids, and proteins; elevated levels of ROS may be linked to mitochondrial dysfunction and associated disorders (32).

## 3 Mitochondrial Dysfunctions and OP

**Figure 2:**
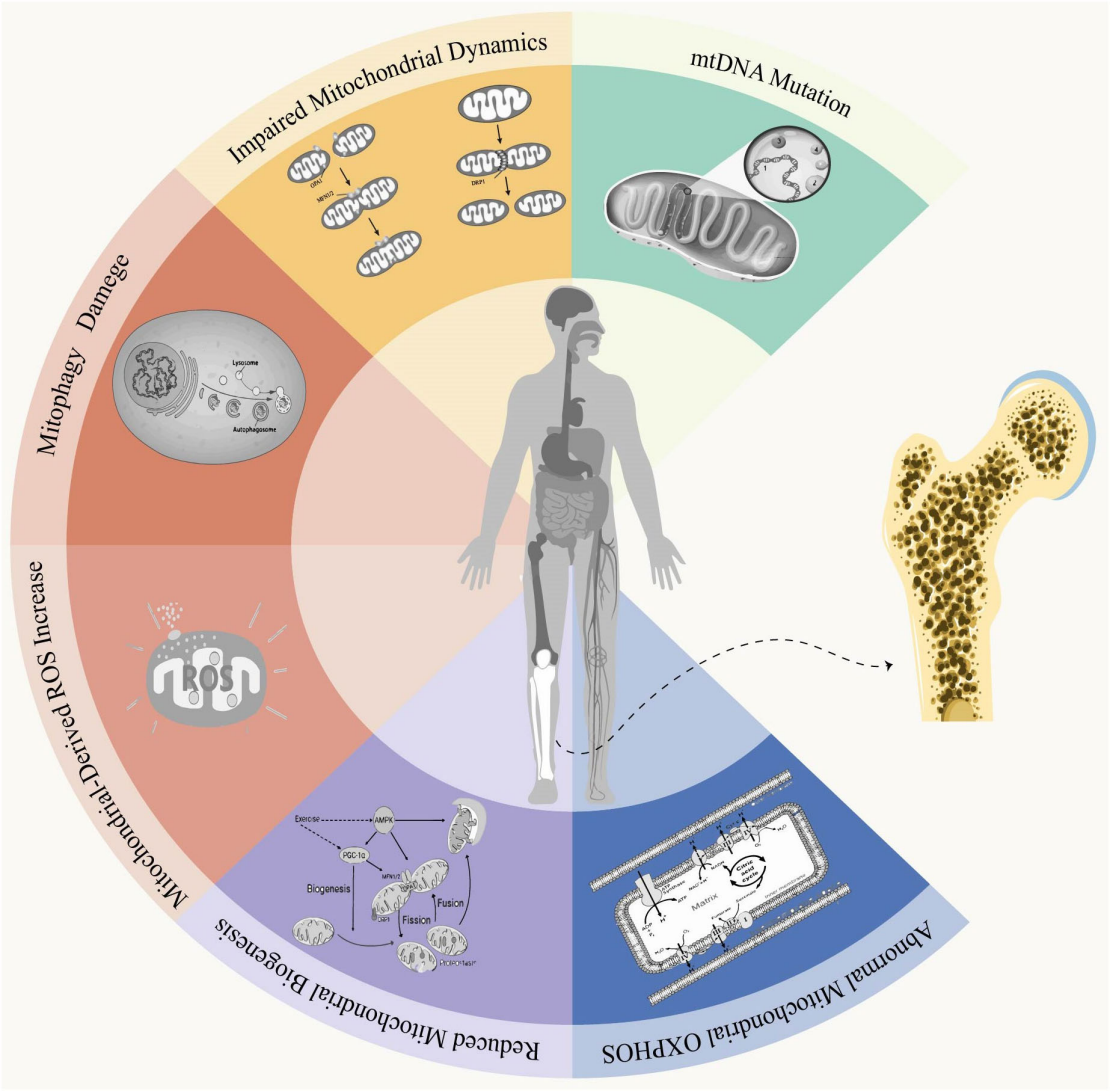
Overview of Bone Homeostasis. Throughout life, bone undergoes constant remodeling and renewal. Osteoclasts are the exclusive cells responsible for resorbing mineralized bone. They adhere to the bone matrix, forming enclosed folded areas before releasing bone-dissolving minerals such as hydrochloric acid and CTSK. Upon completion of resorption, osteoclasts undergo apoptosis. Mesenchymal stem cells (MSCs) progressively differentiate into mature osteoblasts under the influence of relevant factors. Mature osteoblasts synthesize components of the bone matrix, including collagen type 1 (Col1A1), as well as non-collagenous proteins like Osteocalcin (OCN) and Osteopontin (OPN). These synthesized components contribute to the deposition of calcium in crystalline form known as hydroxyapatite deposits, which fill in gaps created by osteoclast activity. Osteoblasts that have completed their role in bone formation either undergo apoptosis or transform into osteocytes embedded within the bone structure itself. Under normal physiological conditions, there exists a dynamic equilibrium between bone resorption and formation processes. However, during aging or under stressful conditions, an imbalance occurs where resorption surpasses formation leading to pathologies such as bone loss.

The pathogenesis of osteoporosis has been continuously researched. Although the precise etiology of OP remains elusive, investigating mitochondrial dysfunction and exploring its association with OP could enhance our understanding and potentially ameliorate this condition. In this study, we provide a comprehensive overview of OP and mitochondrial dysfunction, followed by an in-depth analysis of six key characteristics pertaining to mitochondria in osteoporosis (**Figure 2**).

### 3.1 mtDNA Mutation in OP

Human mtDNA is a circular, double-stranded genome spanning 16,569 base pairs in length. It encompasses 37 genes that play a crucial role in supporting aerobic respiration and cellular energy production through OXPHOS. Unlike nuclear DNA, mtDNA lacks protection from histone proteins and does not undergo recombination events, resulting in an approximately 10-100-fold higher mutation rate (33). These mutations can manifest as point mutations, deletions of fragments or larger-scale rearrangements within the mtDNA sequence, all of which directly impair OXPHOS function (34, 35). Emerging clinical evidence suggests a correlation between mitochondrial DNA mutations and decreased bone mass, particularly in patients with inherited metabolic disorders. For instance, Roshal et al. reported bone loss and fracture in a 16-year-old female harboring a mutation in the POLG1 gene, which encodes DNA polymerase γ responsible for mtDNA replication. De Block et al. documented a case of bone loss and fracture caused by a 7301 bp deletion of mtDNA in a 27-year-old female patient with Kearns-Sayre Syndrome (KSS) (13). Furthermore, a case-control study demonstrated reduced bone mass, cortical thickness, and estimated biomechanics at the lumbar spine, hip, and femoral head among individuals carrying the m.3243A>G mutation. Maternally inherited deafness with diabetes patients carrying the mitochondrial m.3243A>G mutation also exhibit signs of osteoporosis. Additionally, MSCs isolated from individuals with the m.3243A>G mutation displayed mitochondrial dysfunction and impaired osteogenic differentiation (10). In addition, numerous studies have demonstrated that mtDNA mutations are associated with cellular dysfunction (36). For instance, mtDNA polymerase γ (Polg), the sole DNA polymerase present in mitochondria responsible for mtDNA replication and repair across all cell types, exhibits accelerated age-related osteoporosis when mutated (9). Additionally, these mutations impair the ability of osteoblasts to form a mineralized matrix and enhance *in vitro* resorption activity (37). These findings underscore the strong correlation between mtDNA mutations and osteoporosis.

### 3.2 Abnormal mitochondrial OXPHOS in OP

Mitochondrial health is crucial for physiological function, particularly in ATP-demanding tissues. The mitochondrial inner membrane harbors respiratory chain complexes (complexes I, II, III and IV) and ATP synthase complex V, which play a vital role in oxidative phosphorylation (38). Electrons are transferred across the respiratory complexes to generate ATP. Nevertheless, age-related decline occurs in both mitochondrial respiratory capacity and coupling quality control (39). Depletion of the respiratory reserve capacity of mitochondria leads to cellular senescence or death. Bone-forming cells expend significant amounts of energy to sustain their bone-forming capacity and maintain remodeling equilibrium (40). Remarkably, hibernating black bears exhibit an exceptional ability to preserve bone mass throughout the 4-6 months period of hibernation. RNA sequencing analysis of black bears revealed upregulation of the AMPK/PGC1α pathway, which is responsible for mitochondrial biogenesis and respiration. This pathway has been demonstrated to enhance osteoblastogenesis in bone mesenchymal stem cells (BMSCs) (41). Notably, it has been demonstrated that the transition from oxidative metabolism to glycolytic metabolism in aging bones is the primary determinant of bone loss in mice. Stimulation of OXPHOS exhibits an osteoanabolic effect (42). *In vivo*, treatment with sodium oxalate (OXA), a glycolysis inhibitor, resulted in increased trabecular bone density and cortical bone volume in adult mice after 8 weeks. OXA also ameliorated the glycolytic shift observed in the skeletal system of 18-month-old mice, leading to enhanced trabecular bone volume and cortical bone mineral density (BMD) in aged mice. Furthermore, OXA promoted osteoblast-mediated bone formation without affecting bone resorption (43). Similarly, OXPHOS is associated with bone resorption metabolism. Mice exposed to ionizing radiation showed increased bone loss, exhibiting elevated levels of mitochondrial respiration in osteoclasts (44). In addition, it has been shown that the low age-related bone loss in Sirt3-KO mice may be due to reduced oxidative phosphorylation in osteoclasts (45). In summary, mitochondrial oxidative phosphorylation is closely related to bone metabolism.

### 3.3 Mitochondrial-Derived ROS Increase in OP

The mitochondrial ETC and NADPH oxidase (NOX) are closely associated with the production of ROS. During mitochondrial electron transport, complexes I and III serve as the primary sites for ROS generation (46, 47). Through NOX activity, NADPH generates ROS. Mitochondria are abundant in NOX and continuously produce ROS under the combined influence of ETC and NOX (48). Due to their toxic by-products, ROS can impair mitochondria and contribute to the pathological mechanisms of osteoporosis. In turn, damaged mitochondria trigger the release of substantial amounts of ROS from neighboring mitochondria, a phenomenon known as “ROS-induced ROS” (49). Elevated levels of ROS represent one of the pathogenic mechanisms underlying osteoporosis (10, 50). For instance, studies conducted *in vitro* and using mouse models of premature aging have identified mitochondria-generated ROS as a crucial factor in osteoporosis development (9). SOD2-deficient mice exhibit a connective tissue-specific premature aging phenotype accompanied by reduced bone mineral density (51). Other studies have shown that control of H2O2 is a critical part of osteoporosis (52, 53). Production of the intracellular p66shc adaptor protein leads to increased H2O2 production, which is released from the mitochondrial inner membrane inhibitory complex in response to a variety of pro-apoptotic stimuli and reduced to H2O2 by oxidoreductase-catalyzed O2 electron transfer (54, 55). High levels of H2O2 in osteoblasts induce apoptosis and impair osteoblast formation, thereby leading to the development of osteoporosis (56, 57). In contrast, accumulated H2O2 induces osteoclast proliferation and is required for their maturation (58). H202 attenuation contributes to osteoblast formation to promote bone growth through forkhead box O transcription factors (FoxOs) and estrogen regulation (59). The efficacy of these reactive oxygen species defenses diminishes with advancing age (60). Emerging evidence suggests a pivotal role of reactive oxygen species (ROS) in the process of osteoclast formation (15, 61). Initial findings from organ culture experiments indicated the involvement of ROS in osteoclast differentiation and bone resorption, as evidenced by the generation of superoxide anion during osteoclastogenesis induced by parathyroid hormone and interleukin-1, which was attenuated by superoxide dismutase. Subsequent *in vitro* studies have further proposed that elevated levels of ROS may facilitate osteoclast formation, activation, and survival. However, it is worth noting that all previous investigations have established an indirect association between ROS production and either osteoclastogenesis or enhanced bone resorption (58). ROS hyperreactivity disrupts the homeostasis of antioxidants, resulting in an imbalance between bone morphology and resorption (62). Specifically, during youth, the body’s inherent ROS defense system effectively maintains the equilibrium between bone formation and resorption. However, as individuals age, their defensive mechanisms weaken, leading to osteoclasts dominating bone resorption and subsequently causing a decline in bone mineral density and the development of osteoporosis.

### 3.4 Reduced mitochondrial biogenesis in OP

Mitochondrial biogenesis refers to the generation of new mitochondrial mass and replication of mtDNA through the proliferation of existing mitochondria (63). The main regulatory factor of mitochondrial biogenesis, PGC1α, directly controls transcription factors to regulate the expression of nuclear genes. Several transcription factors are required for this progression, including nuclear respiratory factor 1 (NRF1), nuclear factor erythroid 2-related factor 2 (NRF2), peroxisome proliferator-activated receptor-α (PPARα), steroid hormone receptor ERR1 and transcriptional repressor protein YY1 (64). Several studies have shown that mitochondrial biogenesis declines with age over the past few decades. PGC-1α is the master regulator of mitochondrial biogenesis. There is ample evidence supporting the role of PGC-1α in aging. Increased expression of PGC-1α could improve mitochondrial dysfunction and compensate for aging phenotypes in mtDNA mutation mice (65–68). Deficiency of PGC1α, a major regulator of mitochondrial biogenesis, leads to bone loss, as evidenced by: PGC1α deficiency impairing cortical bone mass and bone strength. Moreover, bone marrow precursors of PGC1α deficient mice expressed a lower mRNA level of collagen type I α 1 (Col1a1), the most abundant bone matrix protein (69). Similarly, animals knocked down with SIRT3 had reduced SOD2 expression, inhibited mitochondrial biogenesis, reduced Runx2, Osterix (OSX), and ALP expression, inhibited osteogenic differentiation, and reduced bone mass, while overexpression of SIRT3 improved mitochondrial biogenesis and osteogenic differentiation (70). Abnormalities in mitochondrial biogenesis have also been reported in osteoclasts. It has been shown that sir3 reduces mtDNA content and PGC1-α expression to inhibit osteoclast differentiation and thereby increase bone mass in mice (71). In addition, PGC-β regulates energy metabolism by stimulating biogenesis, and knockdown of PGC-β is associated with promotion of osteoclast differentiation and increased bone mass (72).

### 3.5 Impaired mitochondrial dynamics in OP

Mitochondria demonstrate a remarkable level of dynamism, effectively regulating their quantity, morphology, functionality, and subcellular distribution through the processes of fission and polymerization. Fission facilitates the division of a mitochondrion into two separate entities, while polymerization enables the fusion of two mitochondria together. The hydrolase GTPase plays a crucial role in governing the dynamics of the mitochondrial network. The dynamin DRP1 orchestrates the fission process within mitochondria, whereas MFN1/2 on the outer membrane and OPA1 on the inner membrane mediate mitochondrial fusion (19, 20, 73). When mitochondrial fusion and fission are disrupted, the activity of osteoblasts and osteoclasts is altered, leading to an accelerated onset and progression of osteoporosis. Mitochondrial dynamics play a crucial role in modulating osteogenic processes (74). For instance, senescent BMSCs extracted from individuals with osteoporosis exhibit tubular-shaped mitochondria similar to those found in senescent cells. However, upregulation of miR-21 induces mitochondrial fission, resulting in a decrease in the proportion of tubular mitochondria and an increase in globular mitochondria. Moreover, BMSCs obtained from simple senile osteoporosis patients show down-regulated expression of MFN-1 and MFF (75). Additionally, oxidative stress-induced damage to osteoblasts leads to increased mitochondrial fission that subsequently impairs both mitochondrial morphology and cellular function. Conversely, inhibition or deficiency of Drp1 reduces oxidative stress-induced fragmentation of mitochondria in osteoblasts while mitigating their dysfunction (74). In addition, the modulation of mitochondrial dynamics may play a crucial role in osteoclast-mediated bone resorption. For instance, Ballard et al. developed a murine model with knockout of MFN1 and MFN2 genes in osteoclasts, which resulted in an augmented bone mass and reduced osteoclastogenesis (76). And inhibition of DRP1 suppresses lipopolysaccharide-induced osteoclastogenesis in a cranial model and mitigates ovariectomy-induced bone loss *in vivo (* 77). In summary, perturbations in mitochondrial dynamics contribute to the pathogenesis of osteoporosis.

### 3.6 Mitophagy damage in OP

Mitophagy refers to the selective degradation and removal of senescent or damaged mitochondria by cells in order to maintain mitochondrial homeostasis and ensure the stability of the intracellular environment (78). Among these mechanisms, the PTEN-induced putative kinase 1 (PINK1)/Parkinson’s disease-associated gene (Parkin) pathway plays a crucial role in mitophagy regulation (79). Impairment of mitophagy leads to an accumulation of dysfunctional mitochondria, which may contribute to abnormal bone metabolism and subsequent bone loss. For instance, reduced expression of PINK1 has been observed in patients with osteoporosis, while ovariectomized mice with PINK1 gene defects exhibited significantly decreased bone mass (80). Furthermore, *in vitro* experiments have demonstrated that PINK1 expression increases during normal osteogenic differentiation in osteoblasts. Osteoblasts with low levels of PINK1 expression displayed significantly lower expression levels of osteogenic markers such as ALP, bone sialoprotein (BSP), OCN, and OPN (12). Additionally, these cells exhibited more fragmented and abnormal mitochondria. Similarly, inhibition of mitochondrial autophagy results in abnormal mitochondrial accumulation and excessive production of reactive oxygen species, ultimately leading to mitochondria-dependent apoptosis and impaired osteogenic ability in BMSCs (81, 82). Moreover, studies on Sirt3 have revealed aberrant mitophagy in osteoclasts from mice exhibiting less bone loss (45). Current literature on mitochondrial autophagy in osteoclasts in the context of osteoporosis is limited, necessitating further investigation for more comprehensive insights. Nevertheless, it is undeniable that regulation of mitochondrial autophagy holds promising potential as a therapeutic strategy for addressing osteoporosis.

## 4 Strategies for targeting mitochondria in the treatment of osteoporosis

### 4.1 OPA1

The OPA1 gene, located at 3q28, is a pivotal gene associated with autosomal dominant optic atrophy (83). Mutations in OPA1 lead to the loss of its function, resulting in disease development. This gene consists of 31 exons that encode diverse isoforms. Mitochondrial processing enzymes cleave OPA1 into long(L-OPA1) and short (S-OPA1) forms, which are respectively anchored to the inner mitochondrial membrane (IMM) and the mitochondrial intermembrane space (84, 85). The OPA1 protein has been identified as playing a crucial role in the regulation of mitochondrial dynamics and energy metabolism, as well as maintaining the stability of the mitochondrial genome (86).

The expression level of OPA1 was downregulated in osteoblast apoptosis. Jia et al. demonstrated that cadmium-induced osteoblast apoptosis resulted in mitochondrial dysfunction and reduced expression levels of the mitochondrial fusion proteins OPA1 and MFN2 (87). Similarly, Cai et al.’s study revealed that activation of the AKT-GSK3β signaling pathway by hydroxyl butyl alcohol decreased OPA1 cleavage, effectively maintaining the balance between fusion and fission processes, thereby reducing mitochondrial dysfunction and oxidative stress-induced osteoblast apoptosis (88). Moreover, elevated levels of ROS can induce mitochondrial damage and impair the osteoblastic potential of bone-associated MSCs (89). Chen et al. successfully enhanced superoxide dismutase and catalase activities on implants through a metal-organic skeleton coating with biological functions, leading to ROS decomposition in MSCs, restoration of their mitochondrial function, and significant upregulation of mitochondrial fusion protein expression such as OPA1 and MFN2 (90).

Collectively, these findings underscore the potential role of OPA1 in mitigating or retarding bone loss associated with osteoporosis. The mechanism underlying osteoporosis remains incompletely understood; however, mounting evidence suggests that mitochondrial dynamics plays a pivotal role in this pathological process. Recent investigations have identified the modulation of mitochondrial function as a novel research avenue for treating osteoporosis (14, 91). Several pharmacological formulations and traditional medicines containing biologically active compounds have demonstrated potential in targeting mitochondrial dynamics for the treatment of osteoporosis progression. For instance, hydroxytyrosyl activates the AKT-GSK3β signaling pathway, thereby reducing OPA1 cleavage and maintaining the equilibrium between mitochondrial fusion and fission, consequently mitigating mitochondrial dysfunction and oxidative stress-induced apoptosis in osteoblasts (88). Similarly, silymarin inhibits L-OPA1 cleavage into S-OPA1 and downregulates FIS1 expression in osteoblasts, thus favoring mitochondrial fusion over fission and suppressing advanced glycation end product-induced apoptosis of osteoblasts (92). Although most bioactive substances used for treating osteoporosis are still at the preclinical stage, large-scale clinical trials are warranted to validate their efficacy and safety.

OPA1 plays a crucial role in regulating mitochondrial dynamics by reducing the excessive production of ROS through mitochondrial fusion and timely removing damaged mitochondria via mitochondrial fission, thereby maintaining the integrity of the respiratory chain and stability of mtDNA. Recent studies have highlighted that mitochondrial dysfunction is a significant factor contributing to the occurrence and progression of osteoporosis, while OPA1 has shown promising potential in improving mitochondrial dynamics and preserving mitochondrial morphology and function. Despite some drug molecules and bioactive components being identified for enhancing mitochondrial dynamics and delaying osteoporosis progression, further research on the regulatory mechanism involving OPA1 is still lacking. Insufficient research has been conducted on the impact of OPA1 on bone resorption. Therefore, future investigations should focus on exploring the involvement of OPA1-mediated mitochondrial dynamics in osteoporosis to provide a theoretical foundation for its potential as a therapeutic target for osteoporosis.

### 4.2 PINK

A key regulator of mitophagy is PINK. Under physiological conditions, in a healthy inner mitochondrial membrane, PINK translocates and undergoes rapid degradation (93). However, upon depolarization or loss of membrane potential leading to mitochondrial damage, PINK accumulates on the outer membrane where it phosphorylates Parkin and other outer membrane proteins after recruitment of autophagy machinery, formation of autophagosomes, and subsequent clearance of unhealthy mitochondria (94). Studies suggest that PINK may play a role in the regulation of bone metabolism, potentially through its involvement in the normal regulation of mitochondrial autophagy. Disruption of PINK has been shown to impair mitochondrial autophagy, which can negatively impact osteoblast differentiation and mineralization. Conversely, restoration of mitochondrial autophagy inhibits osteoclast formation and bone resorption, thereby contributing to the mitigation of bone loss (95). Therefore, it is reasonable to hypothesize that PINK-mediated mitochondrial autophagy is implicated in the regulation of osteoporosis occurrence and development.

#### 4.2.1 PINK regulates bone formation

PINK-mediated mitophagy enhances osteoblast differentiation and function, thereby attenuating the progression of osteoporosis (96). Lee et al. demonstrated that PINK deficiency disrupts osteoblast differentiation by perturbing mitochondrial homeostasis (80). The results of tissue staining and uCT analysis in animal experiments demonstrated that PINK1 knockout exacerbated ovariectomy-induced bone loss, while an increased presence of abnormal mitochondria was observed in osteoblasts within the femur of PINK knockout mice. Furthermore, a significant reduction in PINK expression was observed in bone tissue from patients with osteoporosis. In cellular experiments, it was discovered that PINK regulates osteoblast differentiation (80). The beneficial impact of PINK-mediated mitophagy on osteoporosis has also been documented in other investigations. For instance, researchers have discovered that NIPA2 (Prader-Willi/Angelman syndrome region protein 2) deactivates PINK-mediated mitophagy, thereby exerting a positive regulatory effect on the osteogenic capacity of osteoblasts treated with high glucose (97). Similarly, it has been demonstrated that PINK-mediated mitochondrial autophagy inhibits advanced oxidation protein products-induced apoptosis in osteoblasts. *In vitro* studies revealed that rapamycin further activates PINK-mediated mitochondrial autophagy in AOPP-stimulated MC3T3-E1 cells and significantly alleviates AOPP-induced apoptosis by eliminating ROS and damaged mitochondria. *In vivo* studies showed that PINK-mediated mitochondrial autophagy reduces plasma AOPP concentration and inhibits AOPP-induced osteoblast apoptosis, thereby ameliorating bone loss, bone microarchitectural disruption, and bone mineral density loss associated with AOPP accumulation (98). Furthermore, PINK1-mediated mitophagy has been demonstrated to contribute to the production of cathepsin K in osteocytes induced by glucocorticoids. Osteocyte mitophagy may play a crucial role in GC-induced bone loss, suggesting that targeted PINK bone cells mediated by mitochondrial autophagy could be a potential treatment for relieving osteoporosis caused by GC (99).

#### 4.2.2 PINK regulates bone resorption

PINK-mediated mitophagy also exerts an inhibitory effect on osteoclast-mediated bone resorption (100). Wen et al. demonstrated that SIRT3 promotes mitochondrial autophagy in osteoclasts through the deacetylation of PINK, leading to the inhibition of bone resorption. Partial knockdown of SIRT3 impeded osteoclast development and function, resulting in attenuated bone resorption and reduced bone loss associated with estrogen deficiency or aging. These findings suggest that targeted inhibition of Sirt3 can effectively impair osteoclast mitochondrial function via PINK-mediated mitochondrial autophagy, thereby offering a potential strategy for preventing age- and estrogen deficiency-induced osteoporosis (45).

#### 4.2.3 Therapeutic implications

Based on the above introduction, we can conclude that PINK-mediated mitochondrial autophagy ameliorates the progression of osteoporosis by both promoting osteoblast differentiation and function and inhibiting osteoclast formation and/or activity. However, this contradicts the current mechanism of autophagy regulation of bone homeostasis. Specifically, enhanced autophagy contributes to osteoclast differentiation, migration and function, and also to osteoblast activity and function. And there is a lack of directly relevant evidence regarding the role of mitochondrial autophagy on osteoclasts in osteoporosis. Therefore, more studies are needed to clarify the effect of mitochondrial autophagy on the resorptive role of osteoclasts. In addition, besides PINK, there are a number of potential upstream regulators such as Apelin-13, SIRT1, SIRT3, LRRc17, NIPA and MiR-181a that may be involved in the mitochondrial autophagy process (97, 101, 102). Some macromolecular compounds such as resveratrol, laxative salts and vitamin K2 have been reported to modulate mitochondrial autophagy and directly or indirectly improve the osteoporosis condition (103, 104). However, these results are mainly based on animal experiments, and it is not certain whether the same effect can occur in the human body, which needs to be further investigated and verified. Therefore, large-scale clinical trials are necessary to verify whether there is a correlation between mitochondrial autophagy/dysfunction and osteoporosis in humans, and to provide a basis for the development of potential treatments for bone diseases in the future.

### 4.3 Sirtuins

Mammalian sirtuins play a pivotal role in the pathogenesis of age-related diseases and stress response (105). Enhanced sirtuin activity has been associated with reduced susceptibility to age-related ailments, including cancer, cardiovascular disease, and diabetes, as well as potential lifespan extension. These favorable effects may be attributed to the involvement of sirtuins in mitochondrial biogenesis, heightened resistance against oxidative stress, and anti-inflammatory mechanisms. Modulating sirtuin expression or activity holds promise as an innovative therapeutic strategy for preventing and treating osteoporosis (57, 106).

#### 4.3.1 SIRT1

The Sirtuin protein family comprises seven members, namely SIRT1-7, with SIRT1 being the largest in terms of molecular mass and extensively studied (107). Through deacetylating lysine residues on histones or non-histone proteins at both transcriptional and translational levels, SIRT1 achieves epigenetic silencing of key proteins such as p53, FoxOs, and β-catenin (108). Moreover, SIRT1 plays a crucial role in various cellular metabolic processes including tumorigenesis, oxidative stress response, apoptosis, and inflammation. Its ability to resist oxidative stress associated with aging and extend lifespan has earned its recognition as a longevity factor (109). However, there are still significant knowledge gaps regarding the precise mechanisms through which SIRT1 regulates bone homeostasis (110). Earlier studies have demonstrated that knockdown of SIRT1 leads to delayed cortical bone and trabeculae development in young mice, while other studies have indicated that knockdown of SIRT1 results in reduced bone mineral density and an imbalance in osteoblasts/osteoclasts ratio (111).

##### 4.3.1.1 SIRT1 regulates bone formation

The expression level of SIRT1 is associated with bone density and bone fragility. According to clinical studies, the expression of SIRT1 in the femoral neck of osteoporosis patients was significantly reduced. The activity of SIRT1 in peripheral blood mononuclear cells was simultaneously reduced (112, 113). The bone formation ability of SIRT1 knockout mice was found to be diminished, while an increase in bone mass was observed in a mouse model overexpressing SIRT1. Collectively, these findings strongly suggest a close association between SIRT1 and osteoporosis. Moreover, Studies have shown that SIRT1-FoxOs pathways can facilitate osteogenic differentiation by indirectly resisting oxidative stress. According to Jiang, SIRT1 can also deacetylate FoxO1 and promote its nuclear translocation, upregulating the expression of SOD (114). Thus, SIRT1 and FOXOs can scavenge ROS and inhibit diabetes and postmenopausal osteoporosis. And, by reducing ROS in mitochondria and increasing mitochondria biogenesis, SIRT1 can attenuate the inhibition of osteogenic differentiation (115).

##### 4.3.1.2 SIRT1 regulates bone resorption

The anti-oxidative property of SIRT1 inhibits osteoclastogenic differentiation. Through the SIRT1/FoxOs pathway, SIRT1 can reduce ROS levels and inhibit osteoclastogenic differentiation, thus stimulating the expression of FoxOs-mediated catalase and heme oxygenase-1 (116). Oxidative stress can crosstalk with the NF-κB pathway. The NF-κB pathway is activated by oxidative stress through p66Shc phosphorylation. Qu et al. suggested that SIRT1 inhibits the ROS/NF-κB pathway by deacetylating p66Shc, thereby inhibiting osteoclastogenic differentiation (117). Moreover, Feng et al. demonstrated that SIRT1 exerts inhibitory effects on P65 expression in the NF-κB pathway and enhances IκBα expression, thereby effectively suppressing osteoclast differentiation and mitigating bone loss (118).

#### 4.3.2 SIRT3

Sirtuin 3 is a mitochondrial protein that exhibits significant deacetylase activity. It consists of two distinct domains, namely a small zinc finger domain and a large Rossman fold domain responsible for NAD+ binding (119). SIRT3 has been demonstrated to regulate various aspects of mitochondrial function, encompassing energy metabolism, oxidative stress response, mitophagy and beyond (120). For instance, in the presence of hydrogen peroxide, SIRT3 may deacetylate FOXO3, thereby regulating ATP synthesis, maintaining mitochondrial quality, and facilitating damaged mitochondria clearance (121).

##### 4.3.2.1 SIRT3 regulates bone formation

The accumulating body of evidence suggests that targeted intervention towards SIRT3 exhibits promising potential for the treatment of osteoporosis (100). Guo et al. proposed that Sirt3 exerts a protective role against AGEs-induced senescence and SOP of BMSCs. In cellular experiments, they observed that knockdown of Sirt3 exacerbated AGEs-induced senescence in BMSCs by promoting mitochondrial dysfunction induced by AGEs and inhibiting mitochondrial autophagy. In animal experiments, it was discovered that overexpression of Sirt3 had an inhibitory effect on osteoporosis in SAMP6 mice (122). Furthermore, Gao et al. have provided evidence supporting the beneficial impact of Sirt3 on osteoporosis. Specifically, their study demonstrated that the regulation of mitochondrial stress through SIRT3/SOD2 is essential for osteoblast differentiation and bone formation. Notably, mice lacking SIRT3 exhibited significant bone loss accompanied by impaired osteoblast function, whereas overexpression of either SOD2 or SIRT3 enhanced the differentiation potential of primary osteoblasts derived from SIRT3-deficient mice (70). Similarly, Liu et al. have elucidated the distinct role of the SIRT3 S-sulfhydration mechanism in preserving chromatin stability and maintaining mitochondrial homeostasis, thereby impeding the aging process of BMSCs. This discovery highlights a potential therapeutic target for degenerative bone diseases (123).

##### 4.3.2.2 SIRT3 regulates bone resorption

However, several researchers have also demonstrated the detrimental impact of Sirt3 on osteoporosis. For instance, Wen et al. proposed that mitochondrial Sirt3 contributes to age-related or estrogen deficiency-induced bone loss. Their findings revealed that Sirt3-KO mice exhibit reduced mitochondrial respiration and impaired mitochondrial autophagy in osteoclasts, thereby preventing age-related bone loss (45). Similarly, Li et al. demonstrated that deficiency of SIRT3 impeded osteoclastogenesis and mitigated age- or estrogen deficiency-induced bone loss in female mice (71). The authors postulated that the disparate effects of Sirt3 deletion on murine bone phenotypes might be attributed to variations in genetic backgrounds among the mice (45). However, the underlying mechanisms responsible for the disparate effects of Sirt3 on osteoporosis remain incompletely elucidated.

#### 4.3.3 Therapeutic implications

Here we provide a concise overview of the pivotal role played by SIRT1 and SIRT3 in maintaining the delicate equilibrium between bone formation and resorption. In addition to their involvement in mitochondrial regulation of bone metabolism, it is noteworthy that SIRT1 also governs bone vascular homeostasis by promoting angiogenesis while inhibiting vascular calcification. Given the current body of research, targeting SIRT1 emerges as a highly promising strategy for enhancing osteoporosis management. Nevertheless, further investigations encompassing drug trials and animal studies are imperative to unravel the comprehensive impact of SIRT1 on skeletal health. Intriguingly, conflicting findings have emerged regarding the effects of SIRT3. Wen and Li et al.’s work demonstrated that inhibition of sirt3 ameliorated age-related or estrogen deficiency-induced bone loss; conversely, Guo and Gao et al.’s study revealed an exacerbation in bone loss upon sirt3 inhibition but improvement with its overexpression for treating osteoporosis (45, 70, 122). Some researchers hypothesize that these divergent outcomes may be attributed to genetic variations among mouse strains; however, additional studies are warranted to validate this hypothesis (71).

### 4.4mitochondrial-derived peptide

The mitochondrial-derived peptides (MDPs) are translated peptides encoded by mitochondrial DNA genes with short open reading frames (sORFs). It is known that MDPs play a cytoprotective role in preserving mitochondrial function and cell viability during times of stress (124, 125). Two rRNAs, the 12S and 16S rRNAs, and thirteen mRNAs are encoded by the mammalian mtDNA and are involved in the electron transport chain. Eight MDPs are known to exist, and they are all produced from sORFs in mtDNA genes that code for 12S and 16S rRNA transcripts (26). Several MDPs, including humanin (HN), SHLPs, and mitochondrial open Reading Frames (ORFs) of the twelve S-c (MOTS-c), modulate cellular metabolism and provide cytoprotection, changing mitochondrial activity (26, 126).

#### 4.4.1 HN

The mitochondrial polypeptide humanin (HN) was initially identified by Hashimoto in 2001 through screening a cDNA library derived from healthy brain tissue samples of individuals with Alzheimer’s disease (127). HN exhibits anti-apoptotic and neuroprotective properties. It represents the first known example of a mitochondrial-derived polypeptide (MDP) and is encoded by a 75-bp open reading frame (ORF) segment within the mitochondrial 16S ribosomal RNA (rRNA) molecule (128).

Glucocorticoids (GCs) are extensively utilized as immunosuppressive and anti-inflammatory agents in pediatric and adult patients with chronic illnesses (129). Zaman et al. have identified HN as a novel regulator of Hedgehog (Hh) signaling, which effectively prevents GC-induced bone growth disorders while not impeding the expected action of GCs (130). The study demonstrated that overexpression of HN in mice, treatment with HN in wild-type mice, and treatment with HN in cultured rats successfully prevented GC-induced bone growth disorders and chondrocyte apoptosis specifically in metatarsal bones. Additionally, HN exhibited inhibitory effects on GC-induced chondrocyte proliferation. Moreover, it was observed that GC administration reduced the expression of Indian Hedgehog protein in the growth plates of wild-type mice but had no impact on either HN-overexpressing mice or HN-treated wild-type animals (130). Similar investigations conducted on HN have demonstrated that HNGF6A exerts a protective effect against oxidative stress-induced apoptosis in osteoblasts and mitigates the suppression of osteoblast phenotype by modulating the Circ_0001843/miR-214 pathway, as well as downstream kinases p38 and JNK. As an analogue of HN, HNGF6A exhibits cytoprotective and osteopromotive properties in MC3T3-E1 cells, significantly upregulating the expression of proteins associated with the osteoblast phenotype (131). However, the impact of HN on bone loss remains uncertain.

#### 4.4.2 MOTS-c

In 2015, researchers discovered a new MDP called MOTS-c, which follows the discovery of humanin in 2001. MOTS-c colocalizes with mitochondria in a range of tissues and is present in both rodent and human plasma (124, 132). As well as being important for cellular functions, MOTS-c may play a role in hormone regulation.

MOTS-c exhibits several potential applications in the treatment of osteoporosis, including the enhancement of bone density, volume ratio, and cell quantity. The wear-induced osteolysis model of Ultra-High Molecular Weight Polyethylene Particles (UHMWPE) leads to implant loosening and peri-implant fractures by causing bone loss (106, 107). Excessive bone resorption, insufficient bone synthesis, and elevated TNF-α, IL-1, and IL-6 levels brought on by UHMWPE particles are the main causes of bone loss. Interestingly, immunofluorescence assays showed MOTS-c reduced macrophage activity that caused inflammation. Also, MOTS-c injections into UHMWPE particles significantly reduced and reversed bone loss. The study reports that higher levels of TGF-β, SMAD7, and COL1A2 expression were observed at both the mRNA and protein levels in the hFOB1.19 (Human Fatal Osteoblastic) cells treated with MOTS-c (133). Bone organic matter is made up of 80–90% collagen type I (COL1A2). An imbalance between the synthesis and absorption of bone minerals and organic compounds, especially Type I collagen, may cause osteoporosis (134). The findings demonstrated that osteoblasts were stimulated by MOTS-c to produce type I collagen via the TGF-β/SMAD pathway (135). Similarly, MOTS-c treatment increased osteogenic differentiation and TGF-β, SMAD7, and COL1A2 expression in BMSCs at both mRNA and protein levels (136). Additionally, it is also reported that MOTS-c treatment significantly attenuates bone loss in ovariectomized 8-week-old C57BL/6 female mice by inhibiting osteoclast formation in an AMPK-dependent manner (137).

These findings suggest that mitochondria-derived peptides hold promise as a potential therapeutic intervention for osteoporosis. However, several unresolved queries regarding the mechanism of action of mitochondria-derived peptides on osteoporosis necessitate further animal and clinical trials to establish their efficacy.

### 4.4 Mitochondrial transfer

A significant amount of attention is currently being devoted to mitochondrial transfer, a process in which donor cells release mitochondria into the extracellular space for recipient cells to uptake and incorporate into their mitochondrial network, thereby influencing the bioenergy status and other functions of the recipient cells (138). Microvesicles (MVs) and tunneling nanotubes (TNTs) are recognized as the two predominant channels for facilitating mitochondrial transport (139). Donor mitochondria can integrate into the native mitochondrial network of receptor cells through mitochondrial transfer between cells, altering the bioenergy status and other functions of the recipient cells (140).

It has been demonstrated that the distribution of mitochondria in primary osteocyte dendrites declines with age (141). Notably, intercellular mitochondrial transfer also occurs within the osteocyte dendritic network, and transferred mitochondria can restore cellular metabolism in stressed osteocytes lacking functional mitochondria. Moreover, it has been shown that ER-mitochondrial contacts play a pivotal role in mediating mitochondrial transfer between osteocytes, and the involvement of Mfn2 protein is reduced in senescent osteocytes, potentially impacting mitochondrial transfer among these cells (142). Therefore, it is highly plausible that mitochondrial transfer within the dendritic network of osteocytes plays a crucial role in maintaining bone homeostasis and preserving osteocyte viability. Inspired by endogenous intercellular mitochondrial transfer observed during tissue injury to salvage cell or tissue function, we have discovered that artificial *in vitro* mitochondrial transfer to transplanted damaged cells or tissues can effectively repair their damage (143). For instance, recent findings indicate that macrophages present in the bone marrow microenvironment regulate the bioenergetic state and osteogenic differentiation of MSCs by transferring mitochondria into MSCs. Increased transfer of mitochondria from M1-like macrophages (LPS-treated) to MSCs resulted in mice developing symptoms resembling osteoporosis; conversely, transplantation of normal macrophage-derived mitochondria ameliorated bone loss symptoms following OVX surgery (143). Similarly, Guo et al., reported enhanced proliferation, migration and osteogenic differentiation of bone marrow MSCs as well as improved healing of bone defects through mitochondrial transfer therapy (144).

As previously mentioned, mitochondrial transfer from cells has the potential to serve as an efficacious strategy for mitigating mitochondrial dysfunction. However, the application of mitochondrial transfer in the context of osteoporosis still encounters significant challenges that necessitate further exploration, such as donor cell selection, dosage determination, and optimization of cell delivery techniques.

## 5 Conclusions and perspective

Mitochondria play a crucial role in cellular function, encompassing genetic material, energy production, and participation in various metabolic activities. Consequently, any impairment in mitochondrial function can disrupt mtDNA replication, energy generation, and other essential processes, potentially leading to the development of associated disorders. In this study, we primarily investigate the correlation between osteoporosis and mitochondrial dysfunction. Mitochondrial dysfunction in MSCs, osteoblasts, and osteoclasts has been implicated in the pathogenesis of osteoporosis. As our understanding of this disease deepens, we have identified six key features associated with mitochondrial dysfunction leading to osteoporosis: mtDNA mutations, impaired mitochondrial autophagy, reduced oxidative phosphorylation, and increased production of mitochondria-derived ROS. Based on previous descriptions, it is evident that osteoblast mitochondria in osteoporosis undergo a metabolic shift from oxidative phosphorylation to glycolysis, resulting in decreased OXPHOS activity; whereas there is an increase in oxidative phosphorylation observed in osteoclast mitochondria (43–45). Most studies investigating mitochondrial DNA mutations in relation to osteoporosis have primarily focused on patients with inherited metabolic diseases, while other types of studies have been relatively limited. Therefore, further investigations are warranted to establish the association between mitochondrial DNA mutations and osteoporosis. In the literature on mitochondrial autophagy, biogenesis, and kinetic abnormalities in osteoporosis, studies have primarily focused on indirectly substantiating regulatory dysfunctions in osteoporosis by demonstrating increased bone loss through knockdown of a regulatory factor in animal models. However, there is a scarcity of direct evidence showcasing aberrations in mitochondrial autophagy, biogenesis, and kinetics specifically within the context of osteoporosis. Given the predominant reliance on animal and cellular experiments in current research on mitochondrial dysfunction in osteoporosis, it is imperative to conduct more clinical studies to ascertain its relevance to the human condition.

We hypothesize that modulation of mitochondrial dysfunction could serve as a promising therapeutic strategy for improving osteoporosis. In the existing literature, modulation of mitochondrial dysfunction primarily involves mechanisms related to mitochondrial quality control (MQC) (145). Therefore, targeting mitochondrial quality control may offer potential benefits in stabilizing mitochondrial, cellular, and tissue activity and function. Among the various aspects of MQC, extensive research has focused on mitochondrial protein quality control, antioxidant defense systems within mitochondria, regulation of mitochondrial dynamics, promotion of mitochondrial biogenesis, and induction of mitophagy (63, 146–148). Numerous intervention experiments based on animal and human models have been designed by researchers to explore more effective therapeutic strategies for osteoporosis associated with mitochondrial dysfunction. In this review article, we have identified several therapeutic strategies with the potential to modulate mitochondrial function for the treatment of osteoporosis and provided a concise overview of these approaches. Undoubtedly, large-scale clinical trials are warranted in the future to obtain conclusive evidence in patients that can further validate the association between mitochondrial dysfunction and osteoporosis while also facilitating the development of targeted drugs specifically regulating bone homeostasis. Furthermore, recent studies have unveiled a novel mechanism for maintaining mitochondrial quality known as mitochondrial-derived vesicles (MDVs) (149). It can selectively package damaged mitochondrial components into mitochondria-derived vesicles for intracellular transport to lysosomes and peroxisomes for degradation (150). In this way, the structural and functional integrity of mitochondria is maintained. Suh et al. demonstrated *in vivo* and *in vitro* that mitochondria and MDVs secreted by mature OBs can promote osteoblast activity and osteogenesis (151). However, the studies on mitochondria-derived vesicles and bone loss are fewer at present, and more relevant investigations are needed. But undoubtedly, it will also become an effective treatment with the potential to treat osteoporosis.

## Author contributions

JL drafted the manuscript and drew the figures. ZG and XL reviewed and revised the manuscript. All authors contributed to the article and approved the submitted version.
